# Analysis of gut microbiotal diversity in healthy young adults in Sunan County, Gansu Province, China

**DOI:** 10.3389/fcimb.2023.1007505

**Published:** 2023-05-24

**Authors:** Yanqing Ma, Caihong Ci, Yunsong Zhou, Zilong Zhang, Qiaoling Gu, Xiao Yang, Fulong An, Yan An, Yongmei Lan, Jin Zhao

**Affiliations:** ^1^ Medicine Department, Northwest Minzu University, Lanzhou, Gansu, China; ^2^ Department of Surgery, Qinghai Provincial People’s Hospital, Xining, Qinghai, China; ^3^ Department of Internal Medicine, Sunan County People’s Hospital, Zhangye, Gansu, China; ^4^ Key Laboratory of Environmental Ecology and Population Health in Northwest Minority Areas, Northwest Minzu University, Lanzhou, Gansu, China

**Keywords:** Sunan County, Yugur, Han Chinese, microbiota structure, 16S rRNA high-throughput sequencing

## Abstract

**Objective:**

To examine gut microbiotal diversity in the Han Chinese and Yugur populations of Sunan County, Gansu Province, living in the same environmental conditions, and to analyze possible causes of differences in diversity.

**Methods:**

We selected 28 people, ages 18–45 years old, all of whom were third-generation pure Yugur or Han Chinese from Sunan County. Fresh fecal samples were collected, and total bacterial deoxyribonucleic acid (DNA) was extracted. We performed 16S ribosomal ribonucleic acid (16S rRNA) high-throughput sequencing (HTS) and bioinformatics to study the relationships among between gut microbiota structure, genetics, and dietary habits in Yugur and Han Chinese subjects.

**Results:**

We found 350 differential operational taxonomic units (OTUs) in Han Chinese and Yugur gut microbiota, proving that gut microbiota differed between the two populations. That were less abundant among Yugurs than Han Chinese were *Prevotella_9* and *Alloprevotella*. That were more abundant among Yugurs than Han Chinese were *Anaerostipes* and *Christensenellaceae_R-7_group*. And they were significantly associated with a high-calorie diet In addition. we found differences in predicted gut microbiota structural functions (The main functions were metabolic and genetic information) between the two populations.

**Conclusion:**

Yugur subjects demonstrated differences in gut microbiotal structure from Han Chinese subjects, and this difference influenced by dietary and may be influenced by genetic influences. This finding will provide a fundamental basis for further study of the relationships among gut microbiota, dietary factors, and disease in Sunan County.

## 1 Introduction

Numerous microorganisms coexist in the human body. A large number of symbiotic microorganisms are present in the human gut, comprising the gut microbiota. Gut microbiota accounts for 78% of total microbial biomass in the human body and consists of 39 trillion cells, including bacteria, fungi, protozoa, viruses, and archaea. Their total number of genes is 1000× that of the human genome (Yin et al., 2015; Hamilton et al., 2015; Tierney et al., 2019). The human adult gut microbiota is a highly diverse and dynamic ecosystem that is intimately associated with biological activities in the body. Therefore, the gut microbiome is also known as the “second genome” in humans (Zhu et al., 2010). Gut microbiotal structural equilibrium can protect the intestinal mucosal barrier, aid in nutrient uptake, regulate metabolism, promote the maturation of immune tissues, and prevent pathogens from entering circulation; Conversely, when this equilibrium is disrupted, the intestinal mucosal barrier is destroyed, allowing bacterial translocation, gut dysbiosis, and an increase in systemic inflammation, leading to disease (Sonnenburg et al., 2016). The human gut microbiota is influenced by many factors, of which the major ones are genetics, environment, and diet. The latest study by Tavalire published in *mBio (* Koh and Bäckhed, 2020) pointed out that the environment can determine bacterial species in a child’s gut, but genetic factors might determine the abundance of bacterial species therein.

China is a large, multiethnic country with diverse environments. Ethnic minorities in China mostly cluster in various regions, and gut microbiota differ between ethnicities due to the effects of lifestyle habits and genetics (Liu et al., 2019; Liu, 2020; Zhang et al., 2020). There are also significant differences in gut microbiotal structure between ethnicities in certain diseases (Bao et al., 2022; Zeng et al., 2022). Sunan Yugur Autonomous County is located at the Hexi Corridor and northern foothills of the Qilian mountains in Gansu Province, northwest China. The mean altitude of this region is 3200 m, its mean annual temperature is 3.6°C, and glaciers and pastures are present. Ethnic-minority populations that permanently reside in this region have led a nomadic lifestyle for generations, chasing after water and pasture, among them, Yugur is a unique minority in Sunan Yugur Autonomous County, Gansu Province. There are 10684 Yugur ethnic minorities living in Sunan, accounting for 72.65% of the total Yugur population in China (the total population in China is 14,706), and 27.2% of the total population in the county (National Bureau of Statistics of the People's Republic of China, 2022). In order to adapt to a nomadic lifestyle in a high-altitude alpine region, Yugur peoples have developed a culture of unique dietary habits: drinking baijiu, and consuming meat, cheese, shortening, and other high-protein, high-fat, and high-energy food. With the development of urbanization, some of the nomadic life-styles of the Yugur nationality have been transformed into the industrial life-style of urbanization, and have interacted with the Han people living in the area to form similar living habits. The Han nationality is the largest ethnic group in China, and the Han people living in Sunan County account for 42.5% of the county’s total population. Due to low population mobility, the county provides a good sample for studying the influence of dietary habits and genetic factors on intestinal microbiota of different ethnic groups. Previous studies by our group showed that the incidences of essential hypertension, type 2 diabetes, and hyperlipidemia in the region are higher than the national average, and the incidences thereof in the local Yugur population are higher than in the local Han Chinese population (Li, 2016; Gao et al., 2016; Liu et al., 2022; Wei et al., 2022). At present, no study has been performed on the gut microbiotal structure of populations in this region. Therefore, in this study we used high- throughput sequencing (HTS) and bioinformatics to identify the gut microbiotal structural characteristics of Yugur and Han Chinese in Sunan County, as well as to provide new ideas and supporting data for further analyzing the relationship between gut microbiotal structure and chronic non-communicable diseases in different ethnic populations in this environment.

## 2 Materials and methods

### 2.1 Study participants

This study was approved (ID No.: XBNZ-YX-2020058) by the Ethics Committee of Northwest Minzu University (Lanzhou, China). All participants signed the informed-consent form. We randomly selected 28 healthy subjects who were permanent residents (lived there for > 15 years) of Sunan Yugur Autonomous County, Zhangye, Gansu Province; 17 were Han Chinese (HCK), and 11 were Yugur (YCK). Inclusion criteria were as follows: (1) Han Chinese participants must be direct descendants of three generations of Han Chinese. Yugur participants must be direct descendants of three generations of Yugur and follow the traditional lifestyle of the Yugur people.

(2) Participants must be 18–45 years old, and body mass index (BMI) must be 18-25. (3) Abnormalities should be absent from participants’ physical-examination results, and blood biochemistry and blood routine markers should be normal. Participants must not have major illnesses such as cardiovascular, digestive system, or endocrine diseases. (4) Participants must not have taken antibiotics, microbial preparations, or antidiarrheal or weight loss drugs, and must not have a history of diarrhea or other gastrointestinal (GI) diseases, within the last month.

### 2.2 Sample collection

Participants first filled out the basic information and dietary structure questionnaire, and then collected approximately 10g of stool in the morning into a stool storage tube containing stool preservation fluid. The preservation fluid and stool were mixed evenly before the sample was frozen in a - 80°C freezer for ≥24 h. Within 1 week, we shipped samples in dry ice to the laboratory for experiments. (All information, approximately collection and initial storage were completed in the Laboratory of Sunan County People’s Hospital.)

### 2.3 Microbiota DNA extraction and PCR amplification of 16SrRNA fragment

A PowerSoil Deoxyribonucleic Acid (DNA) Extraction Kit (QIAGEN, Hilden, Germany) was used to extract genomic DNA from fecal microbiota per manufacturer’s instructions. We used 1% agarose gel electrophoresis and a NanoDrop2000 spectrophotometer (Thermo Scientific [Thermo Fisher Scientific, Waltham, MA, USA]) to measure DNA concentration and purity, respectively. A suitable amount of sample was added to a centrifuge tube, and sterile water was used to dilute the sample to 1 ng/ul. We used the diluted DNA as a template and specific primers 343F (5’- TACGGRAGGCAGCAG-3’) and 798R (5’-AGGGTATCTAATCCT-3’) with Tks Gflex DNA Polymerase (TaKaRa Bio, Shiga, Japan) for polymerase chain reaction (PCR) amplification of the 16S V3-V4 region in samples to ensure amplification efficiency and accuracy.

The first round of PCR amplification conditions consisted of pre-denaturation at 94°C for 5 min; followed by 26 cycles of 94°C for 30 s, 56°C for 30 s, and 72°C for 20 s; and then a final extension of 72°C for 5 min and holding at 4°C (Murakami et al., 2015). The second round consisted of pre- denaturation at 94°C for 5 min; followed by seven cycles of 94°C for 30 s, 56°C for 30 s, and 72°C for 20 s; and then a final extension of 72°C for 5 min and holding at 4°C.

### 2.4 High-throughput sequencing and bioinformatic analysis

We performed HTS of the PCR amplification product on an lllumina MiSeq system (Illumina, San Diego, CA, USA) to generate paired-end (PE) sequences, also known as raw data. Trimmomatic software version 0.35 (Bolger, Lohse, and Usadel, 2014) was used to remove moving windows with mean base quality <20 from the raw data sequence and sequences <50 bp. We used Fast Length Adjustment of SHort reads (FLASh) software version 1.2.11 (Magoc and Salzberg, 2011) to join PE sequences after removing impurities. The parameters used for joining were as follows: minimum overlap, 10 bp; maximum overlap, 200 bp; maximum rate, 20%. We used Quantitative Insights Into Microbial Ecology (QIIME) split_libraries.py software version 1.8.0 to remove PE sequences containing N bases and retain sequences with a base quality score Q20 of at least 75%. UCHIME software version 2.4.2 (Edgar et al., 2014) was used to remove chimeras from clean tags. Finally, we obtained valid tags for operational taxonomic unit (OTU) classification and used a program written by Shanghai OE Biotech (Shanghai, China) to perform statistical analysis of the entire quality control (QC) process.

Vsearch software version 2.4.2 (Rognes et al., 2016) was used for OTU clustering of valid, good- quality tags obtained from QC based on 97% similarity, and the sequence with the greatest abundance in each OTU was taken as the representative sequence of that OTU.RDP classifier. We used a naïve Bayesian classification algorithm to align and annotate representative sequences with the database. Krona Tools 2.8 (Ondov, Bergman, and Phillippy, 2011) was used to analyze species annotation results, and a Venn diagram was drawn to determine the intersections and unions of differential OTUs between the Han Chinese and Yugur populations. We generated the α-diversity table in QIIME. Alpha diversity is used to analyze species diversity in a single sample. The Simpson, Chao1, and Shannon indices reflect microbiota diversity. With the exception of the Shannon index, the higher the index, the more abundant the species in the sample (Yang et al., 2020) PD_whole_tree reflects phylogenetic diversity and observed species index reflects OTU diversity. R language (Ihaka and Gentleman, 1996) was used to plot a species accumulation curve.

Beta diversity is diversity between habitats, shown by differences between samples from different groups. These differences are usually examined based on OTU sequence similarity or community structure (i.e., species abundance and distribution) or simultaneously based on the phylogenetic relationship of OTU sequences and community structures. The main methods for analyzing β- diversity include distance matrix analysis, principal-components analysis (PCA), PCoA, non- metric multidimensional scaling (NMDS), and unweighted pair group with arithmetic mean (UPGMA). We used a Bray-Curtis dissimilarity algorithm to perform principal-coordinate analysis (PCoA) at the genus level. Based on the taxonomic-analysis results, R was used to plot community composition and relative abundance at the phylum and genus levels in Yugur and Han Chinese samples (phyla or genera with abundance < 0.01 were combined as one group termed others). We employed PCoA and NMDS to analyze differences between our two groups, and analyzed common and specific microbiota of the two ethnicities and studied the dominant microbial structures of different samples.

UniFrac (Lozupone and Knight, 2005) was used to calculate β-diversity, and linear discriminant analysis with effect size (LEfSe) to identify bacterial genera that significantly differed in abundance between the groups at genus level based on an all-against-all strategy. We used the stats package in R and the scipy package in Python to conduct a Wilcoxon rank-sum test of the aforementioned genera in different ethnic samples. The false discovery rate (FDR) method was used to correct multiple tests of *P*-values, evaluate the significance of species abundance differences, and obtain species with significant intergroup differences. We then used these species to study whether species distribution was affected by ethnicity or dietary habits, and we tested significance using the adonis package in R. Phylogenetic Investigation of Communities by Reconstruction of Unobserved States (PICRUSt) was used for function and metabolic-pathway prediction analysis of Han Chinese and Yugur samples, while we used the Kyoto Encyclopedia of Genes and Genomes (KEGG) database to align annotated OTUs in order to obtain functional prediction information. All sequencing and bioinformatics analyses were performed by Shanghai OE Biotech.

### 2.5 Statistical methods

Normally distributed data expressed as mean ± standard deviation (SD; $\bar{X}$ ± *S*), non-normally distributed data expressed as mean and the interquartile (IQR). We performed a *t* test on data with approximately normal distribution and homogeneous variance, and a corrected *t* test on data with approximately normal distribution and heterogeneous variance. A Wilcoxon rank-sum test was performed on non-normally distributed data. Statistical differences were expressed as *P* < 0.05 (*), *P* < 0.01 (**), and *P* < 0.001 (***).

## 3 Results analysis

### 3.1 General information

The study participants were healthy volunteers ages 18-45 years from Sunan Yugur Autonomous County, Gansu Province; 17 were Han Chinese, and 11 were Yugur. The *t*-test result for the mean age of the two ethnicities was *P* = 0.482, and the corrected *t*-test result for the mean BMI was *P* = 0.236. Therefore, there was no statistically significant difference in age or BMI between the two groups (**Table 1**).

**Table 1:** General information of Han and Yugur groups.

Group(N)	Male/Female	Age	BMI
HCK (17)	4/13	34.00±9.31	21.00±2.97
YCK (11)	2/9	39.64±6.10	22.08±1.46
*t*	0.331	0.715	1.215
*P*	0.741	0.482	0.236

HCK, Han healthy young adults; YCK, Yugur healthy young adults.

Yugur Dietary structure analysis showed that the dietary structure of the two ethnic groups was similar, but there was a significant difference in rice and its products, coarse cereals, animal offal, milk and its products, butter and Chinese baijiu products intake between the two ethnic groups. The dietary intake of Yugur was higher than that of Han except for rice and its products (**Table 2**).

**Table 2:** Dietary status of Yugur and Han Mean dietary content (IQR).

Group				
Name of food	HCK(g/day)	YCK(g/day)	Z	P
Wheat and its products	343.4(97.1)	340.7(43.4)	0.588	0.557
Rice and its products***	59.4(15.9)	35.7 (8.5)	4.398	1.09E-5
Coarse cereals***	16.0(5.2)	26.1 (16.4)	4.398	1.09E-5
Potato and its products	11.0(3.4)	10.6 (3.2)	0.542	0.588
Bean and its products	22.5(6.4)	20.3 (7.4)	1.178	0.239
Vegetables and fruits	387.8(55.6)	377.5 (64)	0.635	0.525
Beef and mutton	147.5(37.6)	155.9 (45.2)	0.941	0.347
Poultry meat	30.2(8.6)	26.4 (9.9)	1.812	0.070
Animal offal***	10.4(2.6)	20.7 (3.9)	4.399	1.08E-5
Fried food	11.9(2.9)	13.0 (6.2)	0.377	0.706
Milk and its products**	55.7(19.5)	78.0 (21)	3.624	2.89E-4
Butter***	6.7(1.2)	11.6 (2.6)	4.404	1.06E-5
Edible oil	38.2(9.3)	41.6 (19.8)	0.399	0.711
Chinese baijiu***	23.7(7.0)	39.9(18.0)	4.120	3.78E-5

P values were calculated using the Mann-Whitney-Wilcoxon test (*, P, 0.05; **, P, 0.01; ***, P, 0.001). IQR, interquartile range.

### 3.2 OTU and α-diversity analysis of sample sequences

**Figure 1:**
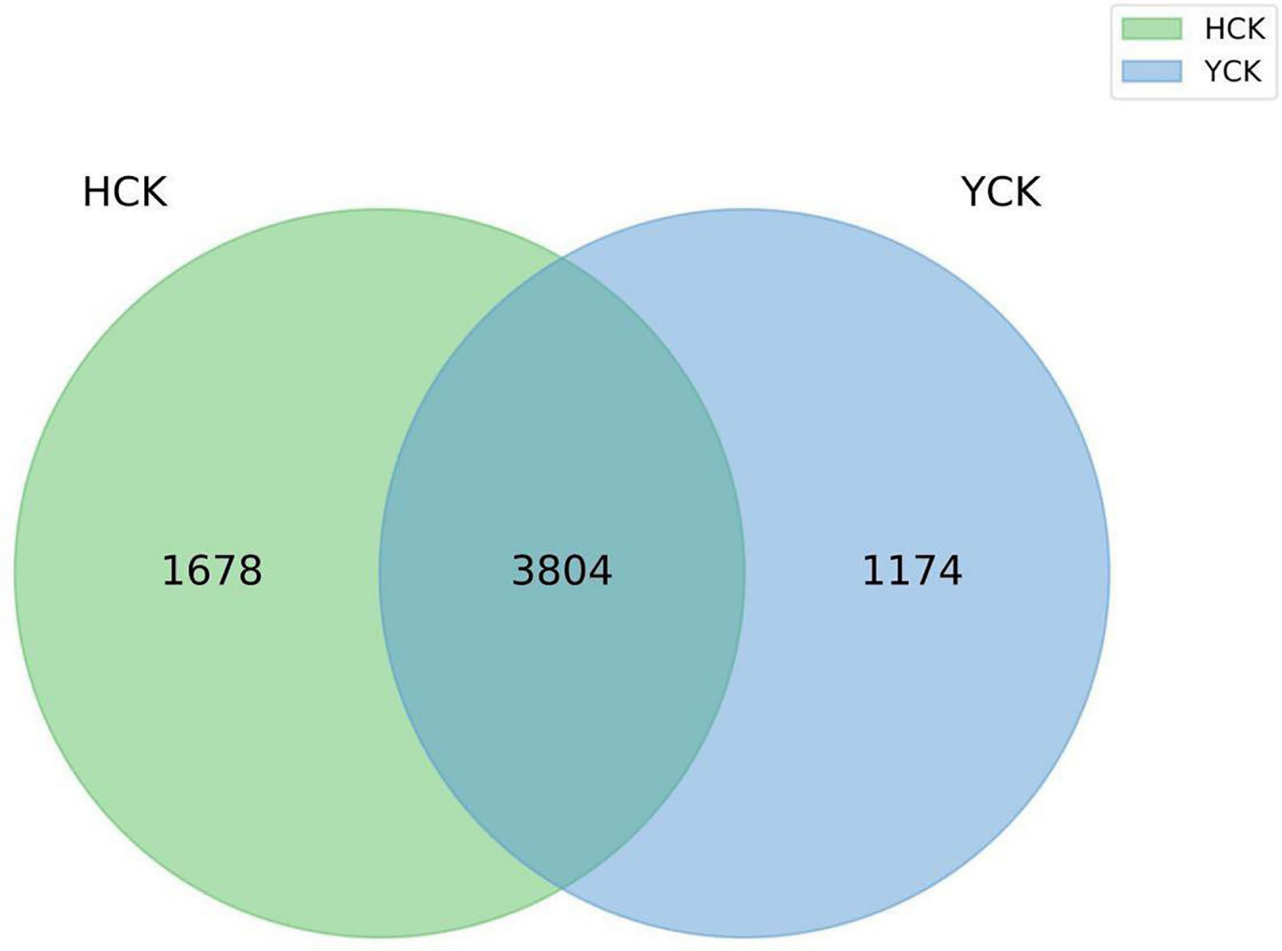
OTU Venn diagram (HCK, Han healthy young adults; YCK, Yugur healthy young adults).

The mean numbers of valid tags in each of the 17 Han Chinese and 11 Yugur fecal samples were 71,461 and 70,057, respectively. We delineated OTUs based on 97% similarity and obtained 6656 OTUs from the two groups of samples. Mean sequencing coverage was 99.17%. There were 1678 Han Chinese-specific OTUs, 1174 Yugur-specific OTUs, and 3804 OTUs common to both groups (**Figure 1**), accounting for 57% of all OTUs. Wilcoxon test analysis found 350 differential OTUs between the two ethnicities.

**Figure 2:**
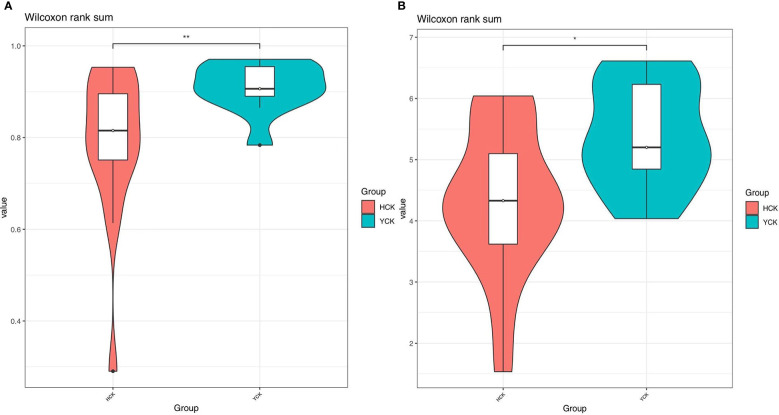
**(A)** Simpson indices alpha_violinplot of differences between HCK and YCK** *P* < 0.01. **(B)** Shannon indices alpha_violinplot of differences between HCK and YCK (*P<0.05; **P<0.01).

**Figure 3:**
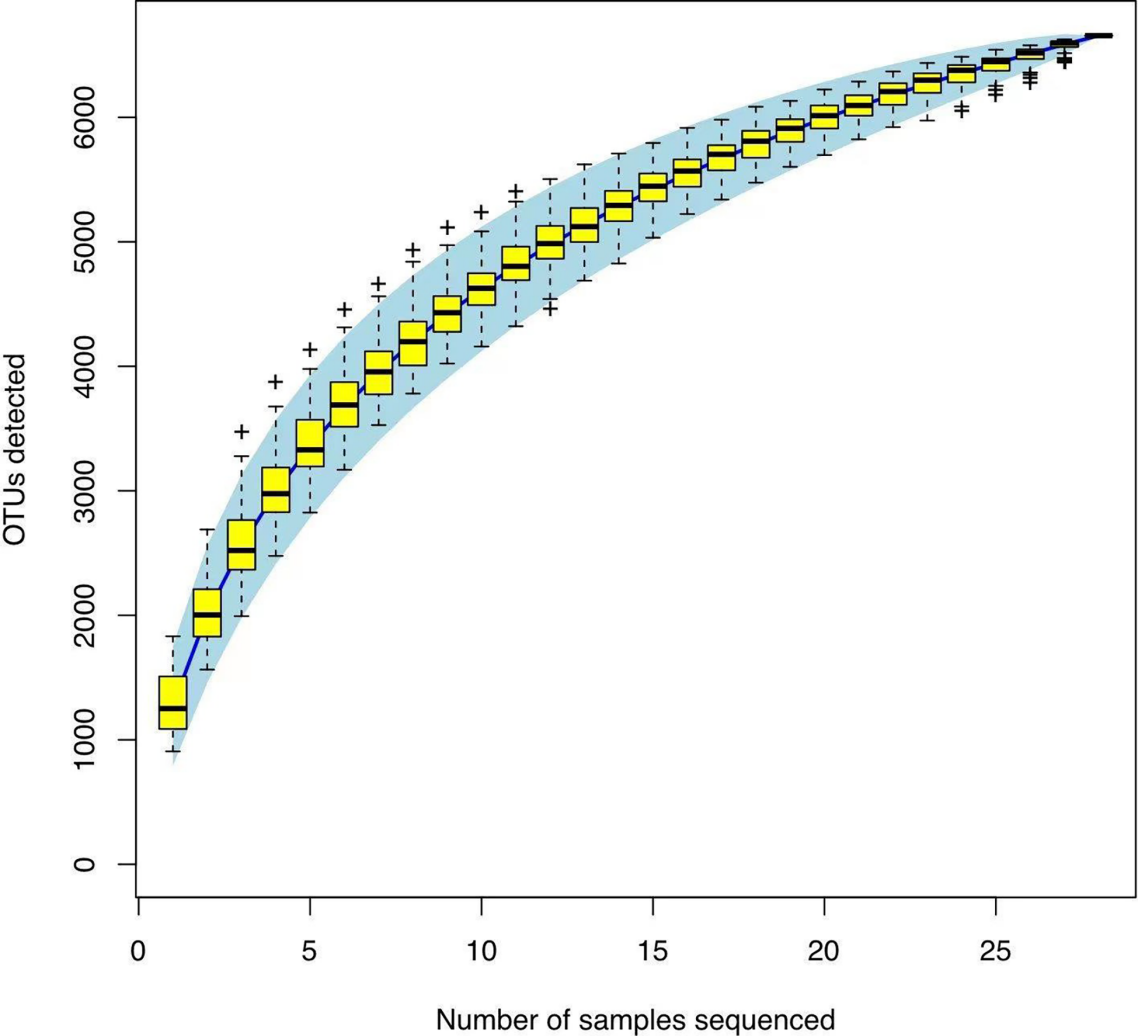
OTU species accumulation map.

In Alpha diversity index, the Shannon index was 4.34 ± 1.22 and 5.40 ± 0.86 in the Han Chinese and Yugur groups, respectively; the Simpson index was 0.79 ± 0.16 and 0.91 ± 0.05 in the Han Chinese and Yugur groups, respectively. Intergroup differences in Shannon and Simpson indices were statistically significant (*P*<0.05; **Table 3**), indicating significant differences in species present between the two groups. The Simpson and Shannon indices of Yugur population was higher than that of Han population, indicating that the gut microbiotal of Yugur population is more diverse sample (**Figure 2A, B**). The species accumulation curve was almost flat (**Figure 3**), showing that the sequencing data volume could reflect microbiota-diversity information in the samples and that the existing sequences satisfied the analysis requirements.

**Table 3:** Alpha diversity analysis indexes of intestinal microbiota in young adults of Han and Yugur.

Group	Observed_species	Chao1	Shannon	Simpson	PD_whole_tree
HCK	1198.59 ± 217.29	1989.73 ± 227.49	4.34 ± 1.22	0.79 ± 0.16	52.70 ± 7.25
YCK	1268.74 ± 287.00	2004.83 ± 259.04	5.40 ± 0.86*	0.91 ± 0.05**	55.33 ± 9.76

HCK, Han healthy young adults; YCK, Yugur healthy young adults; * P < 0.05; ** P < 0.01.

### 3.3 Beta diversity analysis

**Figure 4:**
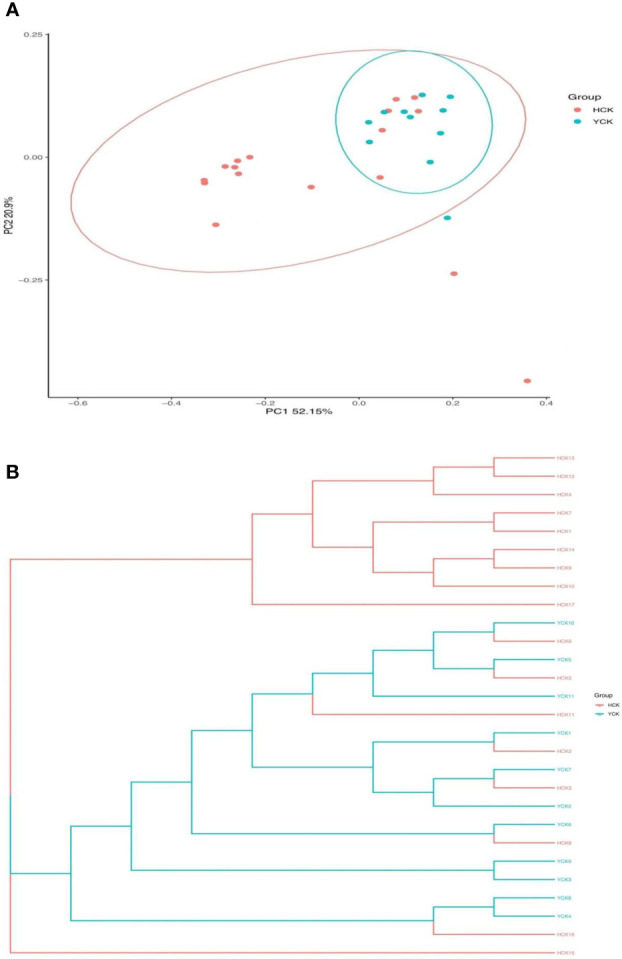
**(A)** PCOA analysis of distance matrix between Han and Yugur groups Note: HCK Han Health Youth Group; YCK Yugur Health Youth Group. **(B)** UPGMA analysis of distance matrix between Han and Yugur groups Note: HCK Han Health Youth Group; YCK Yugur Health Youth Group.

PCoA results (**Figure 4A**) showed that most of the Yugur group samples clustered relatively well, but the Han Chinese groups did not appear well clustered, and six Han Chinese samples clustered with the Yugur group. PC1 explained 52.15% of the intersample variance, and PC2 explained 20.09% of the intersample variance. UPGMA analysis results (**Figure 4B**) showed that were consistent with PCoA results. Analysis of similarity (ANOSIM) showed that *r* = 0.10738 (*P* < 0.05), showing that intergroup differences were greater than intragroup differences in both ethnicities, but little difference between six Chinese Han sample and Yugur sample.

### 3.4 Characteristics and differences in gut microbiota between subjects from the two ethnicities

**Figure 5:**
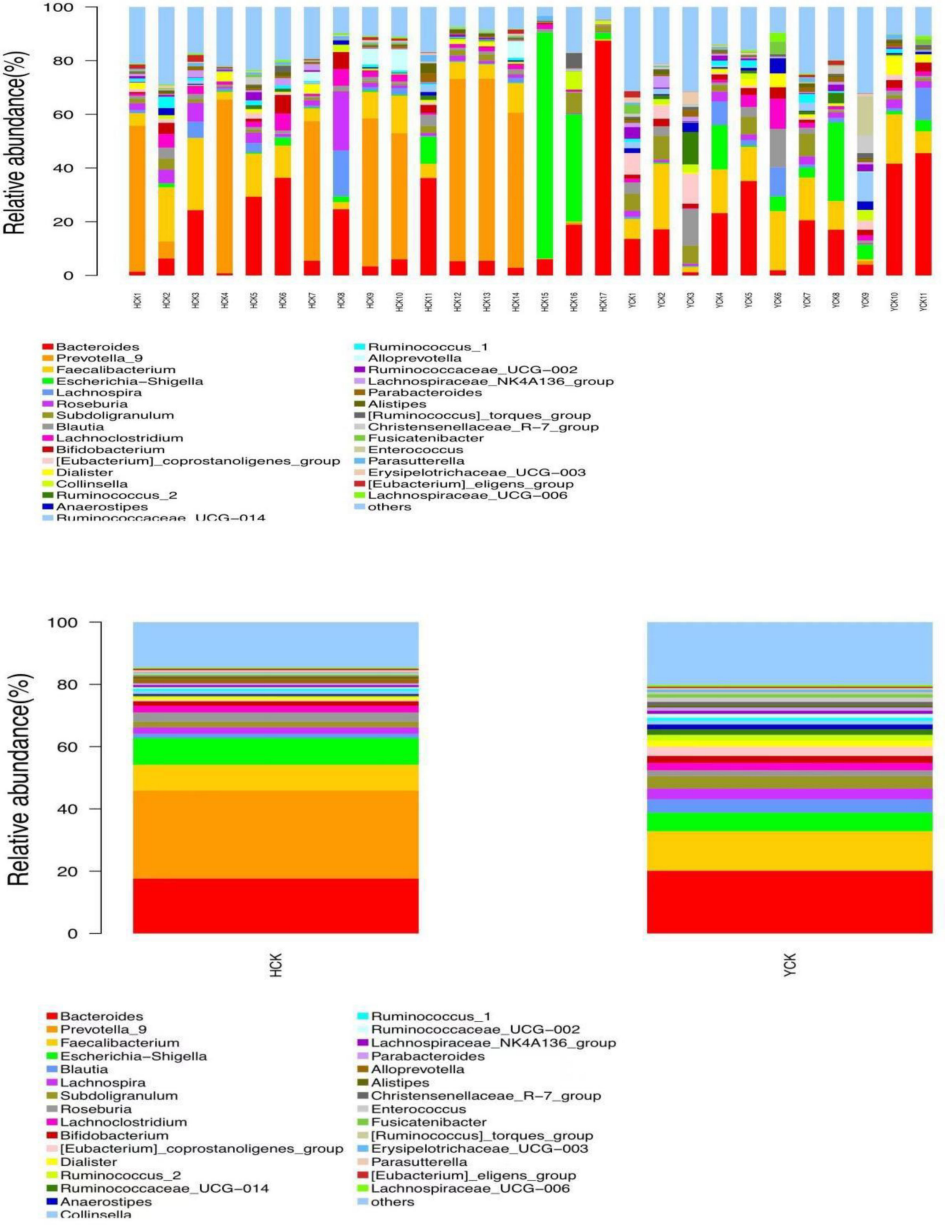
Common microflora of HCK and YCK based on genus level (Top30 abundance).

By comparing our data with the SILVA database (https://www.arb-silva.de/), we found that the obtained OTUs belonged to 29 phylum, 79 classes, 188 orders, 335 families, and 811 genera. Yugur-specific microbiota belonged to phylum Synergistetes and 11 classes, 11 orders, 24 families, and 79 genera; the three highest-abundance genera were *Parvibacter* from the phylum Proteobacteria and *Enorma* and *Varibaculum* from the phylum Actinobacteria, but their abundances were <1%. Han Chinese–specific microbiota belonged to phylum Thaumarchaeota and Calditrichaeota and 6 classes, 20 orders, 34 families, and 102 genera; the three highest- abundance genera were *Ignavibacteriaceae* from the phylum Chlorobi, *Sneathiellaceae* from the phylum Proteobacteria, and *uncultured_Pelobacter_sp* from the phylum Firmicutes, but their abundances were also <1%. Microbiota common to both ethnicities belonged to 26 phylum, 68 classes, 157 orders, 277 families, and 630 genera. Seventeen genera had abundance >1%. The three highest-abundance genera were *Bacteroides*, *Prevotella*, and *Faecalibacterium* (**Figure 5**). The study of bacterial flora with abundance<1% is of little significance, so we mainly explore the characteristics of dominant bacterial flora with abundance >1%.

**Figure 6:**
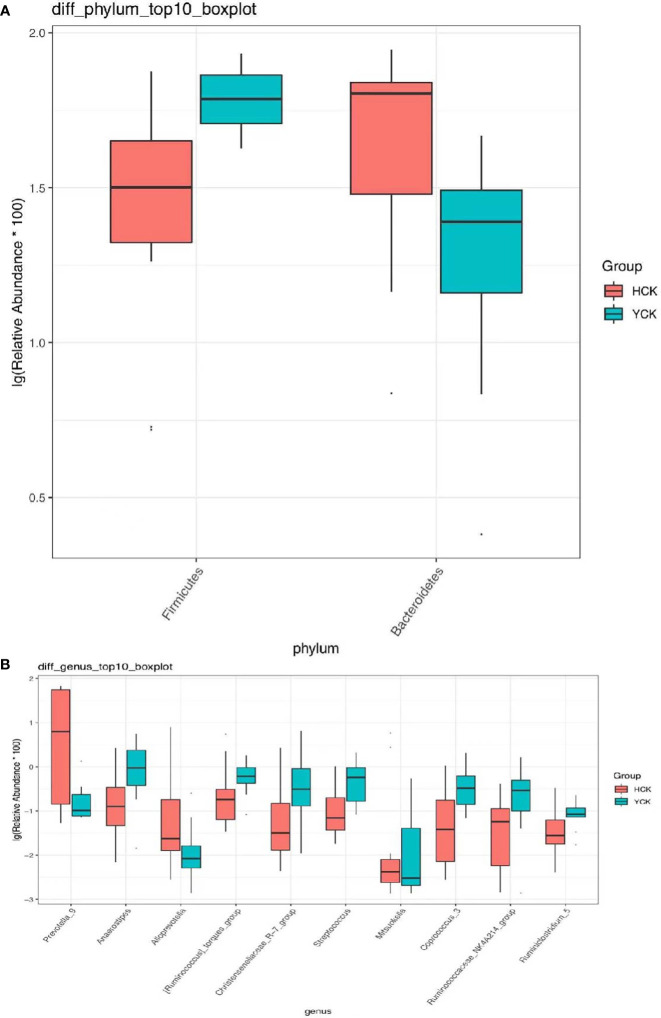
**(A)** Microbiota-phylum box diagram of differences between HCK and YCK (abundance >1%). * *P* < 0.05. **(B)** Microbiota-genus box diagram of differences between HCK and YCK (Top10). *P < 0.05.

**Figure 7:**
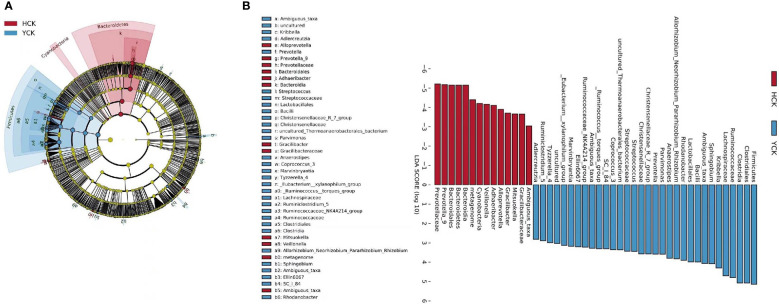
**(A)** LEfSe table cladogram betweenHCK and YCK. **(B)** LEfSe analysis of LDA Score >3 in HCK and YCK.

We used a Wilcoxon test to analyze intergroup differences in microbiota. Results showed 3, 6, 10, 20, and 53 microbiota with significant differences at the phylum, class, order, family, and genus levels, respectively. In the two groups of samples, bacterial phylum with abundance >1% included Firmicutes and Bacteroidetes (**Figure 6A**). Relative abundances of Firmicutes in Han Chinese and Yugur subjects were 34.39% and 61.92%, respectively, with the Yugur group having 1.8× the relative abundance of the Han Chinese group. Relative abundances of Bacteroidetes in Han Chinese and Yugur subjects were 50.74% and 23.95%, respectively, Bacteroidetes’ relative abundance in Yugur group was observably lower than Han Chinese group. The F/B (Firmicutes/Bacteroidetes) ratio was 0.68 in Han Chinese subjects and 2.59 in Yugur subjects, with the Yugur group having 3.8× as the F/B ratio as the Han Chinese group. Genus-level analysis results showed 53 gut microbiotal genera with a difference of *P* < 0.05 between Han Chinese and Yugur subjects, and 4 such genera with an abundance of >1% (**Figure 6B**; **Table 4**). The dominant bacterial genera in Yugur subjects, both from the phylum Firmicutes, were *Anaerostipes*, with an abundance of 1.59%; and *Christensenellaceae_R-7_group*, with an abundance of 1.16%. The dominant bacterial genera in Han Chinese subjects, both from the phylum Bacteroidetes, were *Prevotella_9*, with an abundance of 27.92%; and *Alloprevotella*, with an abundance of 1.41%. The remaining dominant bacterial genera with an abundance of >1% were identical between the groups and mainly from the phyla Bacteroides and Faecalibacterium; the differences were not significant. We used LEfSe multi-level microbiota difference discrimination analysis (**Figure 7A**) to identify species whose abundance significantly differed between the two groups. In the figure, red nodes show bacterial taxa with important roles in the gut microbiota of Han Chinese subjects, while green nodes show bacterial taxa with important roles in the gut microbiota of Yugur subjects. LEfSE analysis found 14 and 23 genera with least absolute deviation (LAD) score > 3 in Han Chinese and Yugur groups, respectively, and the Yugur group had a significantly higher number of microbiota genus than the Han Chinese group (**Figure 7B**). The dominant bacteria in Han Chinese subjects were the *Prevotellaceae* and Bacteroidales from the phylum Bacteroidales, and those in Yugur subjects were the Clostridiales, Ruminococcaceae, and Lachnospiraceae families from the phylum Firmicutes.

**Table 4:** Comparison of the levels of intestinal flora genera with significant differences between Yugur and Han (Top10).

Group				
Name of Bacteria	HCK	YCK	t/Z	P
Prevotella_9	27.931±30.023	0.249±0.357	3.214	0.003
Anaerostipes	0.454±0.741	1.588±1.740	4.573	0.000
Alloprevotella	1.422±2.687	0.041±0.073	2.115	0.041
[Ruminococcus]_torques_group	0.610±1.358	0.722±0.483	4.403	0.000
Christensenellaceae_R-7_group	0.256±0.649	1.163±1.914	3.072	0.004
Streptococcus	0.184±0.251	0.714±0.680	5.015	0.000
Mitsuokella	0.510(0.006)	0.050(0.001)	2.057	0.040
Coprococcus_3	0.144±0.262	0.494±0.597	4.172	0.000
Ruminococcaceae_NK4A214_group	0.091±0.124	0.400±0.438	4.509	0.000
Ruminiclostridium_5	0.060(0.057)	0.095(0.058)	2.234	0.025

### 3.5 Pearson correlation analysis between differential microbiota-genera and differential diet

SPSS Pearson correlation analysis was used to analyze the correlation between the two groups’ diet differences and the difference in microbiota-genera (**Table 5**), four different microbiota-genera with abundance>1% were significantly associated with the different dietary habits of the two ethnic groups. *Prevotella_9 and Alloprevotella* are significantly positively correlated with the intake of rice and its products. *Prevotella_9* has a significant negative correlation with the intake of coarse cereals, Animal offal, butter, and Chinese baijiu. *Anaerostipes* and *Christensenellaceae_R-7_group* are significantly positively correlated with the intake of coarse cereals, milk and its products and butter. This indicates that the difference in gut microbiota between Han Chinese and Yugur is significantly related to diet.

**Table 5:** Summary table of Pearson correlation coefficients between differential microbiota-genera and differential diets and differential diets.

Name of microbiota-genus				
Name of Food	Prevotella_9	Anaerostipes	Alloprevotella	Christensenella ceae_R- 7_group
Rice and its products	0.789**	-0.339	0.445*	-2.71
Coarse cereals	-0.440*	0.445*	-0.26	0.428*
Animal offal	0.541**	0.339	-2.79	0.269
Milk and its products	-0.305	-0.382*	-2.86	0.429*
Butter	0.381*	-0.486**	-1.71	0.366
Chinese baijiu	-0.417*	0.144	-0.340	0.165

P values were calculated using the Pearson correlation analysis (*, P, 0.05; **, P, 0.01; ***, P, 0.001).

### 3.6 16S-based PICRUSt analysis and KEGG functional prediction

We used PICRUSt (Langille et al., 2013) to predict the composition of known microbial-gene functions, thereby calculating differences in functions between different samples and groups (**Table 3**). We found functional differences in 4 kingdoms, 12 phyla, and 97 classes between the two groups.

**Figure 8:**
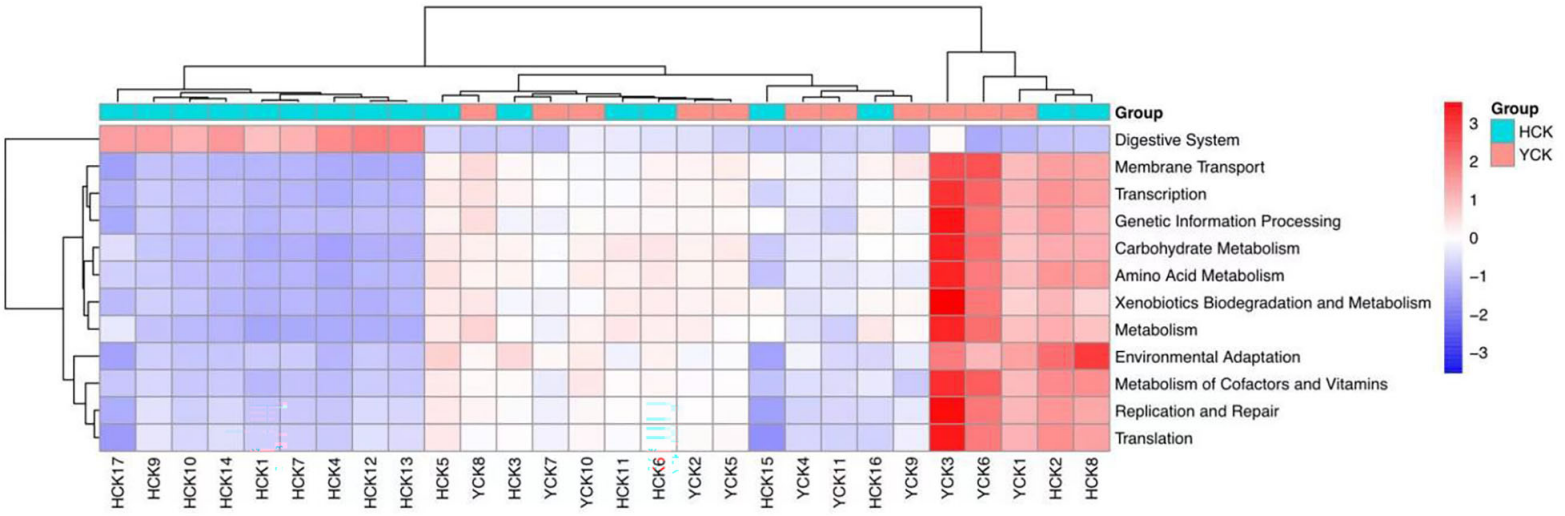
Heatmap of KEGG function prediction based on phylum level in HCK and YCK.

KEGG is a comprehensive database of biological systems and a knowledgebase of related genome and functional information. It consists of gene protein sequences (KEGG Genes), endogenous and exogenous chemicals (KEGG Ligand), molecular-interaction and metabolic-pathway maps (KEGG Pathway), and the hierarchical relationships among various organisms (KEGG Brite). By aligning our results with the KEGG database, we found differences in gut microbiotal functions between Han Chinese and Yugur subjects (**Table 6**). The differential functions mainly included 12 pathways at the phylum level (**Figure 8**), which were digestive system, membrane transport, transcription, genetic- information processing, carbohydrate metabolism, amino acid metabolism, xenobiotics biodegradation and metabolism, metabolism, environmental adaptation, metabolism of cofactors and vitamins, replication and repair, and translation.

**Table 6:** Summary of the functional differences in the PICRUSt.

Level	cog	KEGG_L1	KEGG_L2	KEGG_L3
**Number**	1419	4	12	97

## 4 Discussion

The human gut microbiota is essential to maintenance of host health; its structure is affected by genetics, genes, environment, geography, diet, age, and sex. Recent studies have found that many diseases are intimately associated with gut microbiotal composition, and significant differences in composition exist between different ethnicities (Deschasaux et al., 2020; Bao et al., 2022; Zeng et al., 2022). A 2020 study published in *Nature Medicine* showed differences in gut microbiota among different ethnic minorities and that forced lifestyle changes cause significant changes in these ethnicities’ gut microbiota, which affect health (Sonewane et al., 2022). Research has shown that changes in the gut microbiota of different ethnicities in the same geographical region show ethnicity-specific characteristics and that α-diversity of gut microbiota decreases as metabolic status deteriorates (Tao et al., 2015; Deschasaux et al., 2020). The Yugur are an ethnic minority who lead a nomadic lifestyle in Sunan County, Gansu Province, due to its unique geographical environment. This region is relatively isolated and has low population movement, which makes it suitable for examining the effects of genetic factors and dietary factors on gut microbiota. We selected healthy Yugur and Han Chinese young adults who had lived in this same geographical environment for a long period of time and who had identical lifestyles and similar dietary habits, and we studied differences in gut microbiota between the two groups. We further examined the reasons for these differences based on diet, genetic factors, and the relationship between disease in this region’s populations and gut microbiota to ultimately provide a reference for the diagnosis, prevention, and treatment of certain diseases.

It was found by diversity analysis, although there were six Han Chinese and Yugur samples with similar gut microbiota, there were significant differences in gut microbiotal structure between Han Chinese and Yugur subjects in Sunan County overall. The dominant bacterial phyla in both populations were Firmicutes and Bacteroidetes, which was consistent with the results of a 2018 study by Brookes et al. (Sonewane et al., 2022) that these are the two dominant bacterial phyla in healthy humans. The F/B ratio was 3.8-fold higher in Yugurs than in Han Chinese. The F/B ratio of hypertensive patients is significantly increased (Tao et al., 2015; Lu et al., 2022), and Changes in the he F/B ratio will lead to dyslipidemia (Fan et al., 2021; Mohammadzadeh et al., 2021). Therefore, we speculate that the far higher F/B ratio of healthy Yugur subjects *versus* healthy Han Chinese subjects might be the main reason that the incidence of essential hypertension and dyslipidemia are higher in Yugur than in Han Chinese.

The abundance of bacteria at the genus level, Yugur have 79 specific microbiota- genus, and Han Chinese have 102 specific microbiota- genus, but the abundance of these specific microbiotas- genus was less than 1% and the sample coverage was low, so the significance of the study was small. Further studies with larger sample sizes are needed. We used Wilcoxon rank sum test to analyze the difference between the two ethnic groups. Greater than 1% dominant microbiota- genus were the *Anaerostipes* and *Christensenellaceae_R-7_groups*, which were significantly higher in Yugur than in Han Chinese; *Prevotella* and *Alloprevotella* in Han Chinese population were significantly higher than those in Yugur healthy controls. These four differential bacterial genera were significantly associated with a high-calorie diet. *Prevotella* belongs to the Bacteroides order of the phylum Bacteroidetes, there was a significant negative association with a high-calorie diet and Chinese baijiu intake and a significant positive association with rice and its products. Interestingly, this result is consistent with the most recent dietary and gut microbiota research, a low-calorie diet and a higher intake of rice and its products increase the abundance of Prevotella (Mohammadzadeh et al., 2021; Thwe et al., 2021; Losno et al., 2021). Currently found in metabolic studies of Prevotella, it mainly participates in carbohydrate metabolism, promoting intestinal-nutrient absorption and resisting insulin to improve glucose metabolism (Pedersen et al., 2016; Aslaner et al., 2022). And Prevotella promotes the production of bile acids, which combine with cholesterol to form water-soluble bile salts, affecting the hepato-intestinal circulation of bile acids, thereby reducing the level of total cholesterol in the blood. At the same time, it activates a series of nuclear receptors such as bile acid receptor (FXR) and G protein-coupled receptor 5 (TGR5), which participate in liver bile acid synthesis and intestinal bile acid reabsorption. This reduces the concentration of cholesterol in the body (Liu et al., 2018; Ourakis M et al., 2021). So, we speculate that a high-calorie diet reduces the abundance of Prevotella, which leads to abnormal glucose and lipid metabolism, making the incidence of diabetes and dyslipidemia in the overall Yugur higher than in the Han. The abundance of Alloprevotella was significantly higher in Han Chinese than in Yugur, and there was a significant positive correlation with rice and its product intake. Increased abundance of Alloprevotella reduces blood lipids, but there is no clear mechanism (Liu et al., 2018). Alloprevotella belongs to prevotella genus of Bacteroidetes family of the phylum Bacteroidetes. Probably, its mechanism involved in lipid metabolism is similar to that of Prevotella. Alloprevotella may contribute to the integrity of the intestinal barrier and the suppression of inflammatory responses, improving body immunity (Lijing et al., 2021). The abundance of Anaerostipes and Christensenellaceae_R-7_group was significantly higher in Yugur than in Han Chinese, and there was a significant positive correlation the high-calorie diet. These two bacterial groups were only discovered and defined in the last decade (Sonewane et al., 2022), and they have been less studied.

Current studies have found that they are beneficial to the body’s health and participate in glucose and lipid metabolism (Waters and Ley, 2019; Hul et al., 2020; Bui et al., 2021; Bui et al., 2021).

Through PICRUSt analysis of samples from the Han Chinese and Yugur populations, we were able to predict the functions and metabolic pathways of their gut microbiota. We deduced that 12 functions and metabolic pathways differed significantly between the two groups. Differences in six of the aforementioned functional pathways, such as the digestive system, carbohydrate metabolism, amino acid metabolism, and metabolism, were speculated to be related to dietary habits. Conversely, we speculated that differences in five genetic-information processing pathways were associated with genetic factors, this further verified our inference. This also demonstrated that differences in common gut microbiota between different ethnicities can be caused by many factors, such as dietary structure, environmental exposure, and human genetic variations (Rinninella et al., 2019; Fontana et al., 2019). Regardless of the effects of different factors, changes in gut microbiotal structure and gut microbiotal dysbiosis affect human health. The predictive results for functional pathways in the two groups of samples require metagenomics and metabolomics analysis for further validation.

## 5 Conclusion

In summary, we found significant differences in gut microbiota between healthy Han Chinese young adults and healthy Yugur young adults, and these differences were associated with dietary habits, may be affected by geneticsgenetics. However, further studies are needed to confirm this. In addition, they may be a primary reasons for the high incidence of metabolic diseases in the Yugur population. Although strong evidence correlates gut microbiota with disease, speculations based on differences in gut microbiotal structure between healthy populations are not valid, since ethnicity and dietary habits should also be considered in studies on the correlation between disease and gut microbiota. The results of this study mainly emphasized the differences in gut microbiota between healthy subjects from two ethnicities. They provide a basis for further studies on the correlation between disease and microbiota specific to different ethnicities and on using specific microbial populations for potential diagnosis, prevention, and treatment of health conditions.

## Data availability statement

The data presented in the study is deposited in the NCBI repository, accession number PRJNA971734.

## Ethics statement

The studies involving human participants were reviewed and approved by Ethics Committee of Northwest Minzu University (Lanzhou, China). The patients/participants provided their written informed consent to participate in this study.

## Author contributions

The first author is responsible for data analysis and article writing; The authors ranked second to fifth were mainly responsible for data collection and collation; Authors ranked sixth through eighth were responsible for collecting samples; The corresponding author is responsible for the overall planning guidance.
